# Metagenomic Analysis of the Gut Microbiome of the Common Black Slug *Arion ater* in Search of Novel Lignocellulose Degrading Enzymes

**DOI:** 10.3389/fmicb.2017.02181

**Published:** 2017-11-08

**Authors:** Ryan Joynson, Leighton Pritchard, Ekenakema Osemwekha, Natalie Ferry

**Affiliations:** ^1^School of Environment and Life Science, University of Salford, Greater Manchester, United Kingdom; ^2^Earlham Institute, Norwich, United Kingdom; ^3^Information and Computational Sciences, James Hutton Institute, Dundee, United Kingdom

**Keywords:** CAZymes, lignocellulose, *Arion ater*, biofuel, shotgun metagenomics, whole genome amplification, cellulase

## Abstract

Some eukaryotes are able to gain access to well-protected carbon sources in plant biomass by exploiting microorganisms in the environment or harbored in their digestive system. One is the land pulmonate *Arion ater*, which takes advantage of a gut microbial consortium that can break down the widely available, but difficult to digest, carbohydrate polymers in lignocellulose, enabling them to digest a broad range of fresh and partially degraded plant material efficiently. This ability is considered one of the major factors that have enabled *A. ater* to become one of the most widespread plant pest species in Western Europe and North America. Using metagenomic techniques we have characterized the bacterial diversity and functional capability of the gut microbiome of this notorious agricultural pest. Analysis of gut metagenomic community sequences identified abundant populations of known lignocellulose-degrading bacteria, along with well-characterized bacterial plant pathogens. This also revealed a repertoire of more than 3,383 carbohydrate active enzymes (CAZymes) including multiple enzymes associated with lignin degradation, demonstrating a microbial consortium capable of degradation of all components of lignocellulose. This would allow *A. ater* to make extensive use of plant biomass as a source of nutrients through exploitation of the enzymatic capabilities of the gut microbial consortia. From this metagenome assembly we also demonstrate the successful amplification of multiple predicted gene sequences from metagenomic DNA subjected to whole genome amplification and expression of functional proteins, facilitating the low cost acquisition and biochemical testing of the many thousands of novel genes identified in metagenomics studies. These findings demonstrate the importance of studying Gastropod microbial communities. Firstly, with respect to understanding links between feeding and evolutionary success and, secondly, as sources of novel enzymes with biotechnological potential, such as, CAZYmes that could be used in the production of biofuel.

## Introduction

Slugs are a highly successful group of organisms that are members of the order Pulmonata, found in high abundance in many terrestrial and aquatic ecosystems worldwide. The common black slug, *Arion ater*, is particularly prevalent in Western Europe and North America. These slugs travel long distances at night feeding on a variety of foodstuffs including vegetation (both live and decaying), carrion, and fungi. They use a tongue-like appendage containing barb-like teeth—the radula—to shred their food into uniformly sized pieces, increasing the surface area for enzymatic degradation in the gut. These slugs feed actively down to temperatures approaching 0°C, and adults and eggs have been observed to survive freezing at −3°C for 3 days or more (Slotsbo et al., 2011). It is therefore believed that slugs survive seasonal weather either by preservation of buried eggs or through migration to areas unaffected by frosts, such as, deep in compost heaps and underground in leaf litter (Kozlowski, 2007). Slugs are also known to be resistant to high concentrations of toxic metals, so much so that they are often used in studies of environmental levels of pollution (Ireland, 1979; Seric Jelaska et al., 2014). The ability to utilize a broad range of food sources and their physiological robustness to environmental challenges are amongst the reasons why slugs are such a successful group of organisms, despite the best efforts of humans to eradicate them from agricultural and suburban land.

It is now well established that the gut microbiome plays a pivotal role in digestion in many invertebrates and vertebrates such as, termites (Brune, 2014), cockroaches (Bertino-Grimaldi et al., 2013), cattle (Hess et al., 2011), and humans (Qin et al., 2010). However, the gut microbial communities of members of the gastropod class are still largely unstudied, despite their ability to digest a wide range of materials efficiently. One recent study has demonstrated the ecological richness of the gut microbiome of the gastropod *Achatina fulica* (giant snail), highlighting its metabolic capabilities, with a large number of CAZymes being observed (Cardoso et al., 2012a). In a previous study we demonstrated that the gut microbial consortium of *A. ater* is directly involved in breakdown of the lignocellulose portion of its diet (Joynson et al., 2014), while showing that this enzymatic activity is stable at a broad range of temperatures and pH levels. This suggests that the gut environment of *A. ater* could harbor microbial consortia of considerable ecological and economic importance.

In this study, we examine the composition of gut microbial consortia in *A. ater*, and their metabolic capability. There are three reasons why this research is important. First, knowledge of the gut microbiome composition of *A. ater* offers a means of understanding how this microbial population may facilitate the digestion of lignocellulose along with identification of a large number of CAZymes of interest to many industries including development of second generation biofuels. Second, it may offer insight into the survivability and feeding ability of slug species. This is especially important now, following the European Union ban on traditional molluscicide pellets, in force from September 2014 (Commission Implementing Regulation 187/2014), which was introduced because of the rapid build-up of molluscicide metabolites in water sources (Kay and Grayson, 2013). Finally, the microbiological profile of the slug gut may also provide a target for future bacterial crop pathogen diagnostics, tracking, and control measures in agriculture. Slugs have recently been proposed as vectors for the transmission of bacterial pathogens (Gismervik et al., 2014) and the metabolic capacity of soft rotting pathogens such as, *Dickeya* spp. (identified in this study) and many others could be advantageous in the mollusc gut (Toth et al., 2011).

## Materials and methods

### Sample collection and metagenomics DNA extraction

Slugs were collected from a suburban area in North Cheshire (53.391463 N, 2.211214 W), a sampling area used in a previous study (Joynson et al., 2014), 2 h after last light. Individuals were cooled to 4°C to reduce spontaneous mucus production during dissection. Whole gut tracts were extracted, and care was taken to avoid rupturing the gut wall, to minimize loss or contamination of gut juices. All dissections were carried out in a sterile petri dish. Ten gut tracts were then pooled and DNA extracted using a modified protocol based on the Meta-G-Nome DNA isolation kit (Epicentre, WI, USA). Briefly, gut pieces were homogenized in an extraction buffer by vortexing, and a series of centrifugation steps were then carried out to remove plant material from the gut and other large debris. Supernatants were then filtered through a 1.2 μm filter in order to capture eukaryotic cell debris followed by a microbe capture step using a 0.2 μm filter. Microbes were then washed off the filter and DNA was extracted. DNA quality and quantity was assessed spectrophotometrically (260:230 and 260:280 nm ratios) and using agarose gel electrophoresis alongside a pre-quantified fosmid control. Extracted DNA was then used to create an Illumina DNA library and sequenced using a Miseq using the V2 chemistry (2 × 250 bp) at the Centre for genomic research, Liverpool University.

### Metagenome assembly, functional/phylogenetic analysis

Reads with ambiguous bases, along with their respective pair read, were removed from the raw dataset. Adaptor sequences were then trimmed from raw reads. Sequence output files were assessed using FastQC version 0.10.01 (Andrews, 2010). 25,996,846 reads passed quality control according to FastQC defaults and were assembled using Velvet (V1.2.10) (Zerbino and Birney, 2008) using options: *k* = 51, cov_cutoff = auto, exp_cov = auto and ins_length 200. Velvet output de Bruijn graphs were then used as input to Metavelvet (v1.2.01) (Namiki et al., 2012). To assess the quality of the resulting assembly, raw reads were aligned to resultant contigs using (Burrow-Wheeler Aligner) BWA with default settings (Li et al., 2009). The resulting SAM file was then converted to a.BAM file, sorted, indexed, and mapping statistics obtained using the Samtools (Li et al., 2009) view, sort, index, and flagstat functions respectively. The resulting BAM file was visualized using the TABLET alignment viewer (Milne et al., 2013) facilitating manual curation during selection of novel genes for amplification and biochemical assay. Assembly output contigs were then subjected to open reading frame prediction using the *ab initio* gene prediction method of MetaGeneMark (Zhu et al., 2010). Amino acid sequence files were then used as queries in a BLAST search against the NCBI nr protein database (03/2014) using options: *E*-value cutoff of 1E^−5^, num_alignments 50, and num_descriptions 50 in order to assign putative function. The BLAST alignments were then used to organize predicted proteins into function and phylogeny using MEGAN4 (Huson et al., 2011). The lowest common ancestor (LCA) algorithm of MEGAN4 was used to sort open reading frame alignments into taxonomic groups using default parameters. For functional assignment, the predicted genes were sorted into groups based on the BLAST alignment results and the biochemical pathways annotated in the KEGG database using the KEGG extension in the MEGAN4 software. Further functional assignment was made by searching the predicted proteins against the CAZy database (Lombard et al., 2014). To do this all predicted sequences were used as a query in the CAZYmes Analysis Toolkit (CAT) (Park et al., 2010) using the Pfam based annotation tool with an *E*-value threshold of × 10^−4^. Further phylogenetic analysis was carried out by subjecting raw sequencing reads to analysis using MetaPhlAn V1.7.8 (Segata et al., 2012) incorporating BowTie2 (Langmead and Salzberg, 2012). Raw reads were also uploaded to the MG-RAST pipeline (Meyer et al., 2008) for functional and taxonomical assignment along with estimation of taxonomic abundance. SEED analysis was used to compare the functional repertoire of slug gut microbiome against public MG-RAST gut metagenomes for higher termites (Costa Rican *Nasutitermes* sp.) cattle (*Bos taurus*), the Asian longhorn beetle (*Anoplophora glabripennis*) and the giant African land snail (*A. fulica*). In order to gain insight into biologically meaningful and statistically significant differences between the functional capacities of the slug gut and other microbiomes, the two-way Fisher's exact test with Benjamin-Hochberg FDR multiple test correction analysis was carried out pair wise between SEED annotations of the slug gut microbiome and those of comparator organisms using Statistical Analysis of Metagenomic Profiles (STAMP) (Parks et al., 2014). *A. ater* sequencing data was submitted to EBI ENA database (project ID: PRJEB21599).

### Amplification, cloning, and expression of CAZymes

To increase the amount of metagenomic DNA template available for metagenome validation and amplification of identified genes, metagenomic DNA from the same sample that was used in sequencing was subjected to whole genome amplification (WGA). Ten nanogram of metagenomic sample DNA was used as template for amplification using the Repli-G mini kit (Qiagen, Manchester, UK), producing 4–6 μg of whole genome amplified product per 10 ng starting material. In order to validate the metagenomic assembly a selection of predicted CAZY gene sequences were amplified using 100 ng of WGA metagenomic DNA as template using Taq based PCR. PCR products were separated using 1% agarose gel electrophoresis and bands of sizes corresponding to the size of the predicted genes were gel extracted. PCR primer sequences and predicted genes sizes can be found in Supplementary Dataset 3. Amplified bands were then cloned and transformed into *E. coli* using the TA cloning kit (pCR2.1 vector) (Invitrogen). Vector inserts were sequenced using the BigDye 3.1 system to confirm CAZyme identity. One full length gene was subsequently re-amplified using Taq based PCR and cloned into the pBAD TOPO TA expression vector (Life Technologies, Paisley, UK). Proteins were expressed according to the manual instructions, and expressed products assessed using western blot targeting a C terminal His-tag. Detection was carried out using a secondary antibody-HRP conjugate and the ECL prime chemiluminescence kit (GE healthcare, Buckinghamshire, UK).

### CAZymes activity detection

To detect enzyme functionality, transformed strains expressing proteins were then grown on agar activity assay plates. Strains containing predicted β-glucosidase cloned pBAD TOPO TA expression vectors were induced as per manual instructions. Five micro liter of induced culture was grown on LB agar plates containing 0.1% (w/v) of the cellobiose mimic, esculin hydrate (Sigma, UK), and 0.03% (w/v) ferric ammonium citrate (Sigma, UK) for 24 h. The production of black halos was taken to indicate β-glucosidase activity. Untransformed TOP10 *E. coli* was used as a negative control.

## Results

### Metagenomic library sequencing

Metagenomic DNA isolated from the whole gut tract, including crop and stomach was successfully extracted and the purity and genomic integrity tested as described. Sequencing of the metagenomic DNA yielded over 6 Gbp of raw sequence data in the form of ~26 million paired-end reads, with an average length of 238 bp. The resulting community metagenome contained 81.74 Mbp of sequence data with assembled contigs having an N50 value of 1.8 Kbp (Table 1). This metagenome was then mined to determine the gut community ecology profile, along with the functional and metabolic capabilities of the microbiome.

**Table 1:** Sequencing and assembly statistics of the gut community metagenome.

	***A. ater* Gut Metagenome**
Number of trimmed reads	25,996,846
Raw sequence data (Gbp)	6.175
Number of assembled contigs	48,089
Largest contig (Kbp)	56.3
N50 value (Kbp)	1.8
Protein coding genes	108,691
Total size of metagenome (Mbp)	81.74

### *A. ater* gut microbial diversity

**Figure 1:**
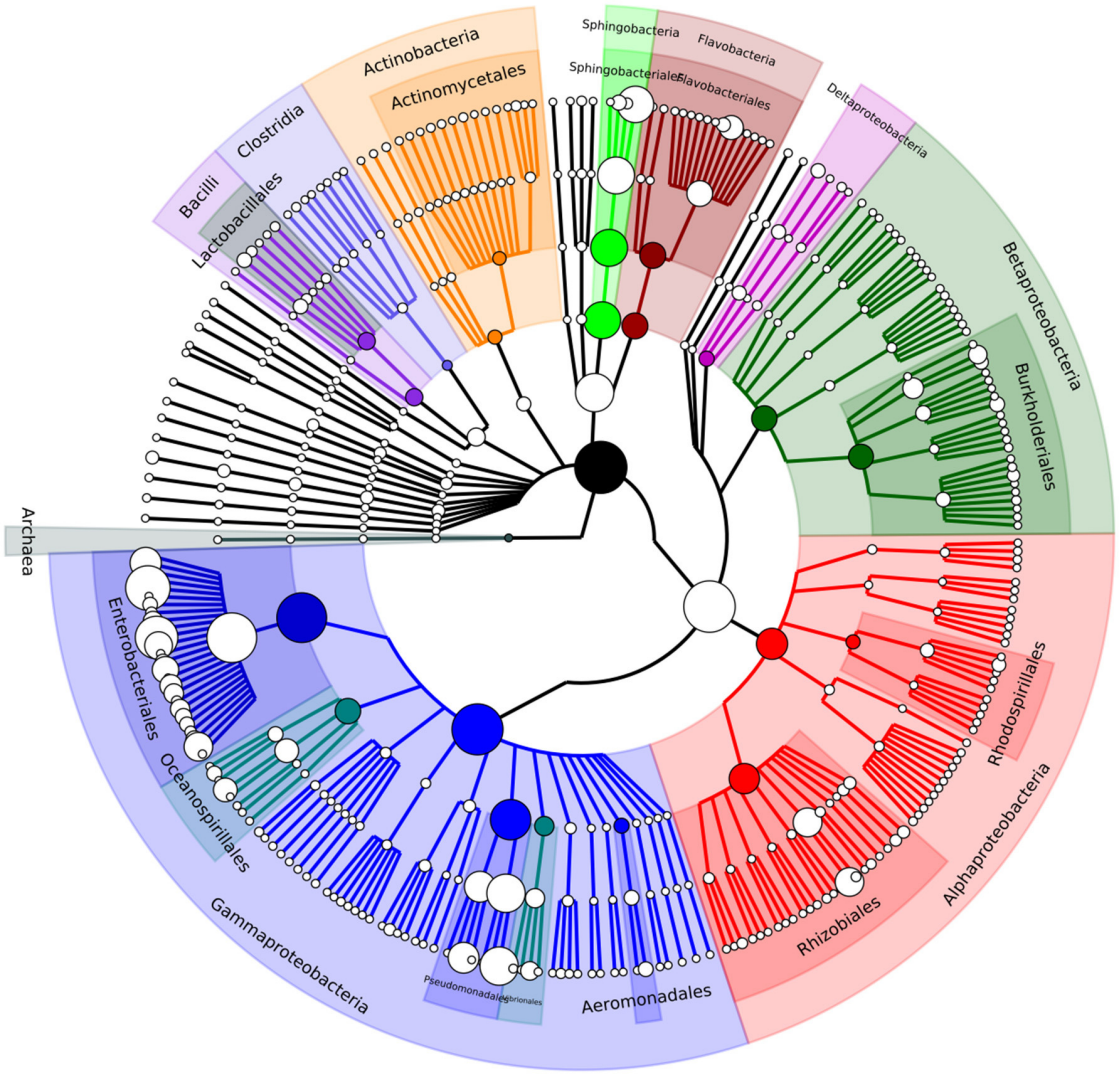
A phylogenetic tree showing the diversity of the *A. ater* gut microbiome down to genus level. Visualised using GraPhlAn (Asnicar et al., 2015).

Metagenomic community analysis showed that bacterial DNA predominated in the sample, with 99.4% of reads corresponded to bacteria, and only 0.3% to viruses, 0.2% to eukaryotes, and 0.01% to archaea (Supplementary Dataset 1). This suggests that attempts to limit the number of host and plant DNA contaminants by filtering was highly successful. Relative abundance of microbial groups was assessed using MetaPhlAn. This analysis indicated that the majority of the gut microbial community corresponded to members of the Gammaproteobacteria class (82%) with most assignments being to members of the Enterobacteriaceae (64.5%) and Pseudomonadaceae (10.6%) families, which both contain widespread environmentally-adapted bacteria. Other families with notably high representation in the gut were Sphingobacteriaceae (8.6%), Moraxellaceae (3.7%), and Flavobacteriaceae (1.8%). The most abundant genera found in the gut microbiome were *Enterobacter* (26.9%), *Citrobacter* (19.8%), *Pseudomonas* (10.5%), *Escherichia* (3.9%), and *Acinetobacter* (3.6%), and the genera *Pantoea* (2.7%), *Klebsiella* (2%), *Serratia* (0.75%), *Erwinia* (0.73%), and *Salmonella* (1.1%) were also identified at lower abundance (Table 1, Figure 1, Supplementary Dataset 2). In order to compare the assignments and abundance data generated here, reads were also submitted to the MG-RAST pipeline which uses global alignments in its analysis unlike the marker gene database system used by MetaPhlAn. The MG-RAST pipeline produced results comparable to those from MetaPhlAn; again, the Gammaproteobacteria class was by far the most numerous in the sample, with the majority of those hits matching the Enterobacter family (Supplementary Dataset 1).

### Presence of potential plant pathogens

To determine the presence of plant pathogen species harbored in the *A. ater* gut, the metagenome phylogenetic analysis results were mined for hits relating to known plant pathogen species using the phylogenetic analyses of MataPhlAn and MG-RAST. Multiple assignments of metagenome sequence to plant pathogenic bacteria could be made, and **Table 3** shows six economically significant plant pathogens identified in the *A. ater* gut microbiome. These include the three most economically-damaging bacterial crop pathogens in Europe: *Erwinia amylovora, Dickeya dadanttii*, and *Pectobacterium carotovorum*. (The species in **Table 3** were identified by both MetaPhlan and MG-RAST analysis methods).

### Functional analysis and bacterial metabolic processes

**Figure 2:**
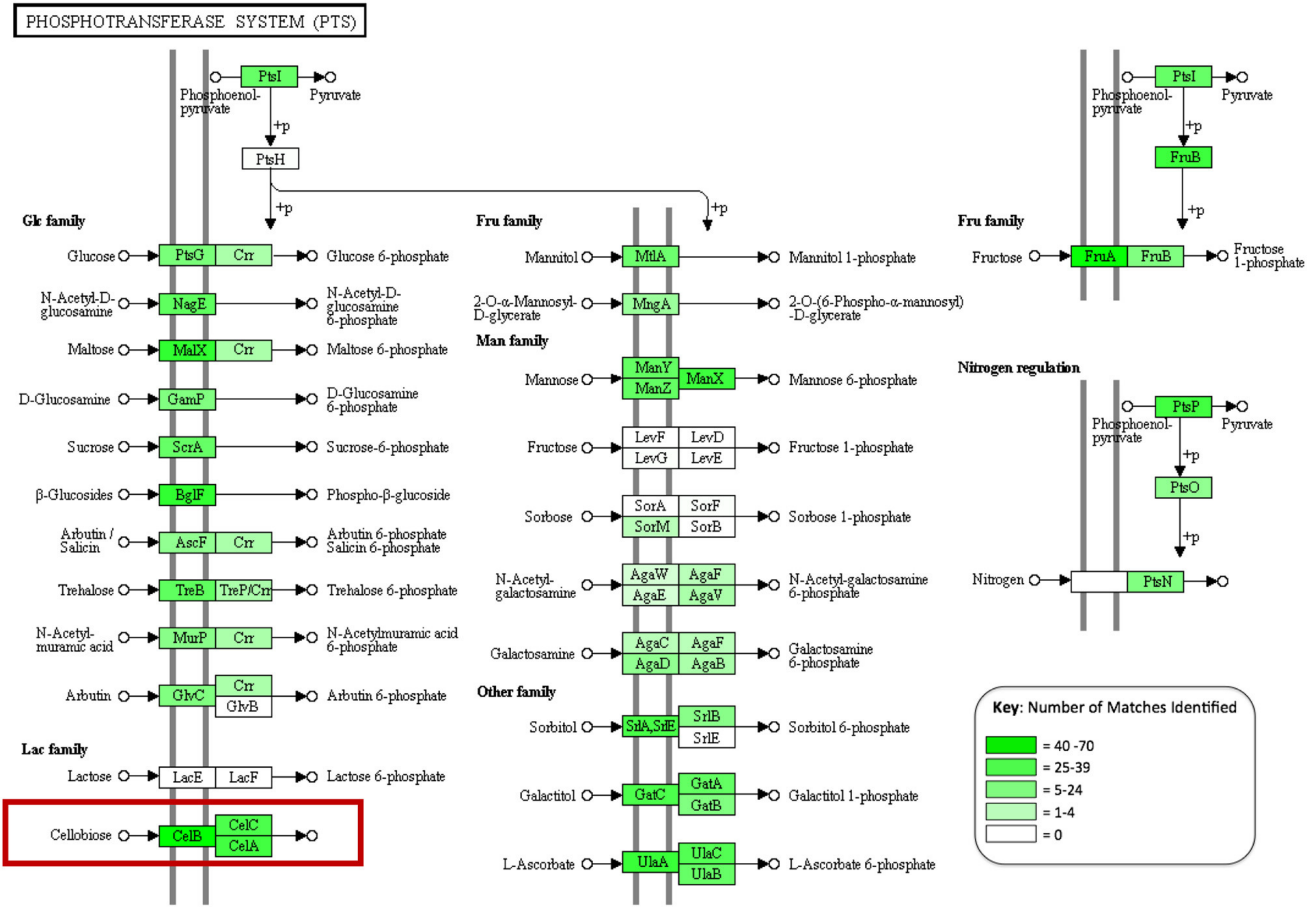
A KEGG diagram showing the phosphotransferase system (PTS), genes identified in the gut metagenome are highlighted in green with color intensity corresponding to abundance observed (created in MEGAN4).

In order to assess the biochemical/metabolic potential of the gut microbiome, genes were predicted from assembled contigs. In total 108,691 putative genes were identified. These predictions were translated into amino acid sequences and used as queries for protein family identification, based on hits to the CAT Pfam database. This search identified 2,510 genes corresponding to glycoside hydrolase activity and 561 carbohydrate-binding modules. The majority of the carbohydrate-active genes identified were assigned to enzyme groups that break oligosaccharides down into simple sugars (641, 20.8%), with fewer targeting cellulose (26 enzymes, 0.85%) (**Table 4**). This search also identified 312 members of the relatively new CAZyme classes “Auxiliary activities” or AA classes, which describes enzyme classes that act on or consort with lignin in their activities (Levasseur et al., 2013). This included 150 members of the class AA3, 2 members of AA2, 11 members of AA4, which are involved in the oxidative degradation of lignin, and 60 members of class AA6, which catalyze reductive degradation of aromatic compounds such as the monolignols that make up the lignin superstructure. Predicted protein sequences were also subject to BLAST analysis against the NCBI non-redundant (nr) database using BLASTp. In total 97,882 predicted proteins were matched to sequences in the nr database (~90% of total predictions). Using the KEGG extension of MEGAN4, over 32,000 functional associations were made to KEGG biochemical pathways from the BLAST output, of which 8,333 were attributed to carbohydrate metabolism. Multiple assignments to phosphotransferase systems (PTS) that facilitate internalization of many sugars in bacteria were also observed (Figure 2). These included 109 proteins that make up the three subunits of the PTS that facilitates specific internalization of cellobiose.

**Figure 3:**
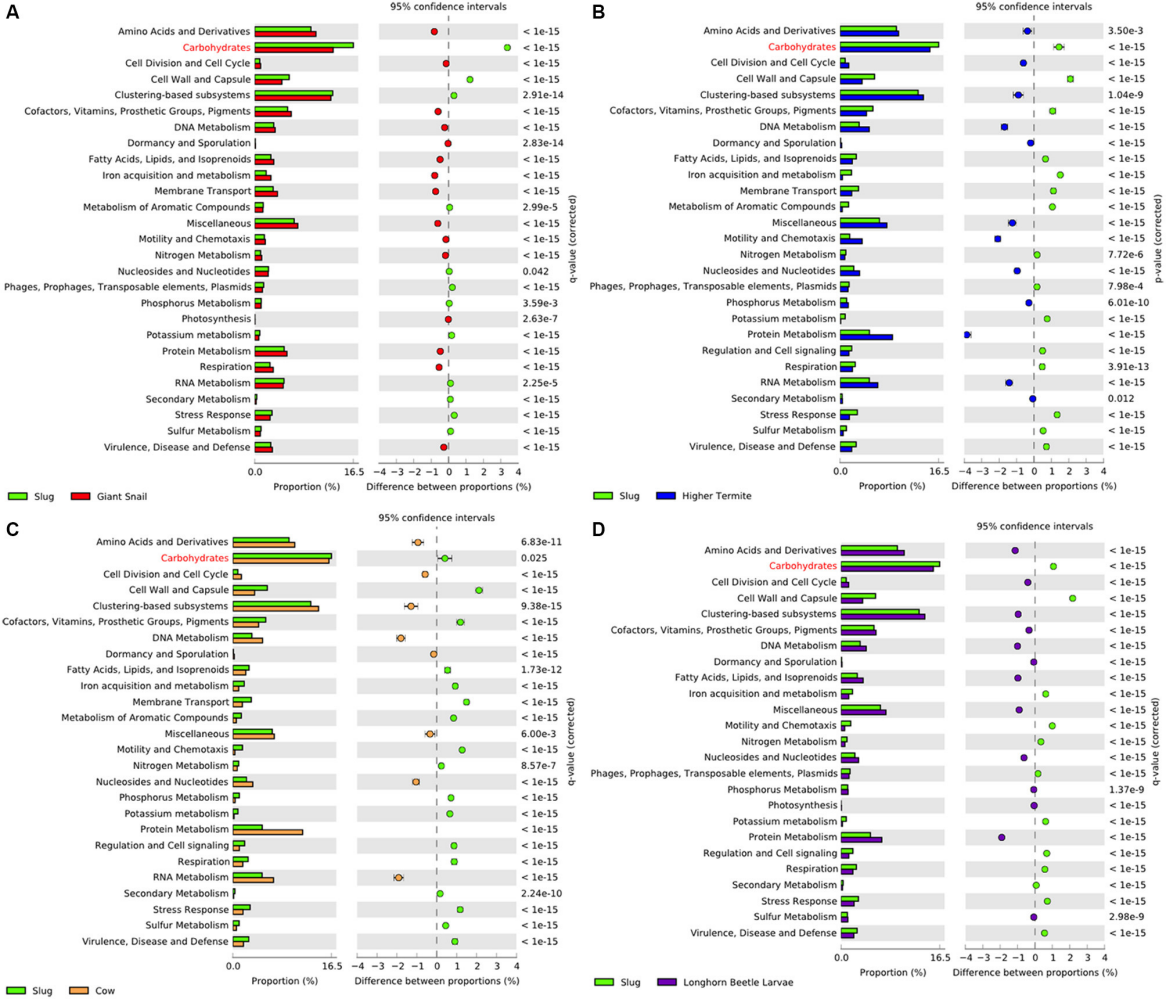
Extended error bar percentage representation plots of SEED functional groups in the *A. ater* gut compared to other gut metagenomes. Pair-wise comparisons were made for the *A. ater* metagenome against **(A)** giant snail, **(B)** termite, **(C)** cow, and **(D)** long horn Asian beetle gut metagenomes.

The gut CAZyme profile generated for *A. ater* was compared with those of humans, termites wallabies, giant pandas, and giant snails (**Table 4**). This comparison demonstrates that the number and proportion of cellulase-degrading enzymes in the slug gut are similar to what is found in both the snail and wallaby, with a similarly high number of oligosaccharide degrading enzymes in both molluscs. However, in the slug gut environment many more enzymes targeting hemicellulose were identified than in any of the comparator organisms. The SEED functional classifications of the microbiome were also compared to those of other gut environments, which demonstrated an increase in the proportion of genes involved in the processing of carbohydrates in the slug gut than in any comparator environment (Figure 3). This comparison also revealed that the SEED group representation in *A. ater* and the giant snail (*A. fulica*) gut metagenomes were much more similar to each other than to the mammalian and insect comparator gut environments (Figure 3).

### Amplification and expression of CAZymes

**Figure 4:**
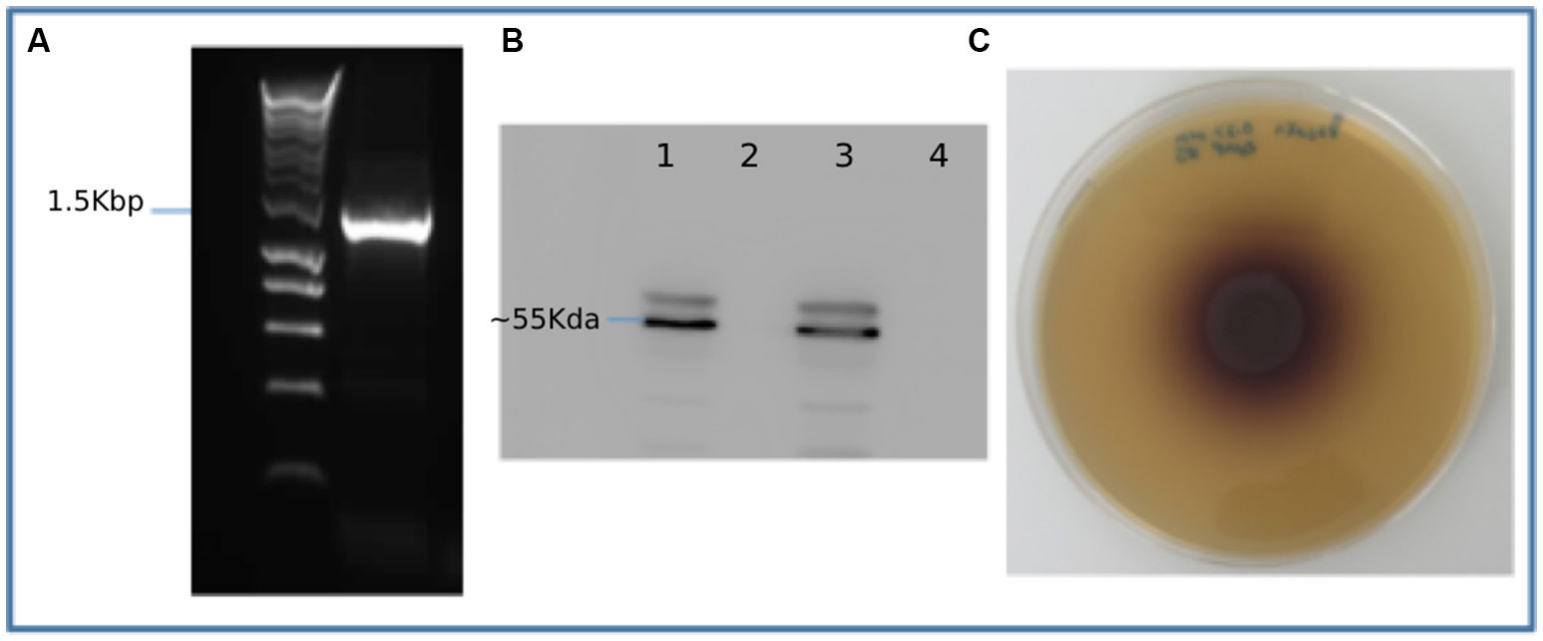
Recombinant expression and activity testing of gene 9459: **(A)** Amplification of gene 9459 **(B)** A Western blot showing successful expression of recombinant protein lanes 1 and 3 showing duplicate induced samples and lanes 2 and 4 showing duplicate negative controls **(C)** An esculin hydrate- ferric ammonium citrate activity plate showing the gene 9459 clone β-glucosidase activity.

To validate the metagenomic assembly and gene predictions, multiple genes were selected for amplification from the original metagenomic DNA sample. These included two full length predicted endocellulase genes, a full length β-glucosidase gene, a full length xylanase gene and a full length FAD-linked oxidase from the auxiliary activities 4 CAZyme group (Supplementary Datasets 3–5). As a proof of principal one partial gene was amplified (gene_id_77908) and subsequently extended to a full length gene using primers designed based on the top BLAST hit for that specific gene. Sanger sequencing of the resulting amplicons was carried out confirming amplification of the targeted predicted gene sequence. Five of six genes targeted were successfully amplified and full sequences confirmed. Gene 9459, a predicted β-glucosidase was also successfully amplified (Figure 4A), cloned and expressed in *E. coli*. The expression of a recombinant His-tagged protein of predicted size (~55 KDa) was confirmed using Western blotting (Figure 4B). The 9,459 strain was grown on a β-glucosidase activity growth plate (Figure 4C) and tested positive for β-glucosidase. A control of untransformed TOP10 *E. coli* showed no activity on this assay.

## Discussion

The common black slug, *A. ater* has become one of the most widespread and successful Gastropod species in Europe and North America. The success of this (and other) species has caused the UK agricultural industry alone to spend almost £30 million each year on molluscicide pellets (Agular and Wink, 2005). Making it an important species in agro-economical terms.

Research into the digestive system of *A. ater* began in the 1960s, focusing on both carbohydrate breakdown (Evans and Jones, 1962) and protease activity (Evans and Jones, 1962). Further work determined rates of cellulose breakdown and characterized the pH and temperature profiles of gut fluids from black slugs of North American origin (James et al., 1997). In a previous study, we characterized the biochemical activity in the gut of the British black slug and identified multiple gut bacteria that exhibit cellulolytic activity. This work implicated the gut microbiome in the degradation of plant cell wall into simple sugars. In this study we tested the hypothesis that the slug gut microbiome could contribute to digestion and nutrient cycling, especially the breakdown of complex plant cell wall superstructures that are notoriously difficult for animals to degrade without substantial assistance from microbes (Hansen and Moran, 2014). This study has revealed an ecologically rich consortium of bacterial species in the *A. ater* gut that have previously been implicated in the digestion of tough vegetation. We have also demonstrated the vast metabolic repertoire that exists within the slug gut microbiome, including enzymes with potential to contribute to degradation of every major component of plant cell wall superstructure, including lignin, which is widely considered to be the most difficult of these compounds to degrade enzymatically (Sanderson, 2011).

In total, *Gammaproteobacteria* accounted for the vast majority of the community metagenome, with 82% relative abundance; this included identification of 84 species in this class. The most abundant genera identified include *Enterobacter, Citrobacter, Pseudomonas, Eschericia, Acinetobacter*, and an unclassified genus belonging to the *Sphingobacteriaceae* family. These genera alone accounted for almost three quarters of the sequenced component of the gut metagenome (Table 2). Previous studies have shown dominance of the phylum *Proteobacteria* in gut microbiomes of various gastropod species, including freshwater planorbid snails (*Biomphalaria pfeifferi*) and terrestrial snails such as, the giant African land snail (*A. fulica*) (Cardoso et al., 2012b). *Proteobacteria* have also been seen to dominate other insect gut microbiomes whose diets are largely or entirely comprised of lignocellulose (Dillon and Dillon, 2004; Russell et al., 2009), which suggests a general association of this phylum not only with herbivorous insects but also with plant-eating gastropods. Furthermore, two studies of microbial consortia in fungal gardens used by leaf cutter ants (*Atta colombica*) to degrade lignocellulose both report dominance of the family *Enterobacteriaceae* (which account for ~65% of the *A. ater* community metagenome) and predict this family to be directly involved in the efficient breakdown of plant material in these gardens (Suen et al., 2010; Aylward et al., 2012). A large number of genera were also detected in much lower abundances with over 200 genera account for only ~27% of the microbiome, these may comprise transient elements of the gut microbiome that are ingested during proximal feeding or suppressed by nutritional cycling in the gut at a particular time. Our findings are also consistent with previous culture dependent identification of cellulolytic microbes from the *A. ater* gut, where almost all identifications made were in the *Gammaproteobacteria* class, and included many of the more abundant genera noted in this study (Joynson et al., 2014). These findings suggest that the gut environment of *A. ater* contains a consortium that is reflective of many highly efficient lignocellulose degrading environments.

**Table 2:** A selection of the most abundant phylogenetic groups present in the gut microbial community down to genus level.

**Classification**	**Percentage abundance (%)**
k__Bacteria	99.99
k__Archaea	0.01
p__Proteobacteria	88.15
c__Gammaproteobacteria	82.16
o__Enterobacteriales	64.56
f__Enterobacteriaceae	64.56
g__Enterobacter	26.86
g__Citrobacter	19.86
g__Escherichia	3.91
o__Pseudomonadales	14.25
f__Pseudomonadaceae	10.56
g__Pseudomonas	10.54
f__Moraxellaceae	3.69
g__Acinetobacter	3.68
p__Bacteroidetes	10.53
c__Sphingobacteria	8.57
o__Sphingobacteriales	8.57
f__Sphingobacteriaceae	8.56
g__Sphingobacteriaceae_unclassified	8.10
p__Firmicutes	0.59
p__Actinobacteria	0.28
p__Chlamydiae	0.21
p__Chloroflexi	0.16

*Phylogenetic classifications and microbial abundance estimations were made using MetaPhlAn to compare sequences to a clade specific marker database*.

Mining of the phylogenetic data associated with the gut microbiome identified several bacterial plant pathogens. These included six species recently ranked among the top 10 most important species of plant pathogen (Mansfield et al., 2012; Table 3). Many of these pathogens are known to cause necrosis and eventual development of soft rot, blight, or blackleg in tuber based crops such as, potatoes, but also in ornamental plants and other crops. These include the three relatively closely related *Enterobacteria Dickeya dadantii, P. carotovorum*, and *E. amylovora* (Toth et al., 2011) with the latter two being identified previously in *A. ater* gut from samples taken in 2012 from the same area as this study (Joynson et al., 2014). If both of these pathogen species are commensally present in the slug gut, this would suggest that *A. ater* may act as a perpetual vector species through which they could be spread from field to field, and persist between growing seasons by overwintering in the slug gut. The role of insects in the transmission and overwintering of plant pathogens is now quite well established, the squash bug, flea beetle, and cucumber beetle are known to spread plant pathogens as well as sustaining populations of the pathogens they harbor during dormant winter months (Nadarasah and Stavrinides, 2011). However, more indepth study over multiple seasons would be required to confirm this hypothesis.

**Table 3:** Microbiome abundance of plant pathogens present in the *A. ater* gut microbiome, as ranked by a survey of experts carried out by Mansfield et al. (2012).

**Ranking**	**Pathogenic species**	**Microbiome abundance (%)**
1	*Pseudomonas syringae*	0.08264
3	*Agrobacterium tumefaciens*	0.06987
5	*Xanthomonas campestris*	0.0144
7	*Erwinia amylovora*	0.03587
9	*Dickeya dadantii*	0.04896
10	*Pectobacterium carotovorum*	0.04215

Functional analysis of the *A. ater* metagenome has yielded identification of 3,383 genes involved in the degradation of plant biomass, including all of the major components of the plant cell wall superstructure, cellulose, hemicellulose, and lignin supporting previous work that has implicated the slug gut microbiome in the facilitation of lignocellulose degradation (James et al., 1997). The largest proportion of these (641) breakdown oligosaccharides, including 204 β-glucosidases, 80 β-galactosidases, and 279 β-xylosidases. Numbers of long chain carbohydrate degrading enzymes were lower in comparison, with only 26 cellulase enzymes being identified in total. The dominance of oligosaccharide degrading enzymes appears in all of the other comparator gut environments shown in Table 4, including wallabies, termites, and also in the gut microbiomes of reindeer and cattle (Pope et al., 2012) with similar patterns also observed in environmental microbiomes such as, leaf cutter ant fungus gardens (Aylward et al., 2012). This could support the hypothesis that gut microbes are predominantly involved in the breakdown of partially degraded plant material (be it partially rotten when ingested or chemically pre-processed in a stomach) across the board. However, there is still the possibility that some groups of microbial lignocellulose degrading enzymes that are unknown and may be undetectable using homology-based methods. Enzyme groups that are involved in the degradation of hemicellulose are seen in especially high numbers in the *A. ater* gut when compared with other gut microbiomes, with larger numbers for both the degradation long chain hemicellulose (321) and its derived oligosaccharides (437). Further indications that sugars in plant cell walls are utilized by gut microbes come from the identification of numerous sugar transporter proteins. These include a large number of components of the cellobiose-specific PTS that facilitate the uptake of cellulose degradation products (Figure 2). The KEGG diagram in Figure 2 also shows the presence of membrane transport system components specific to mannose and β-glucosides. Together, the identification of multiple enzymes that break down plant cell walls and the transport systems that facilitate the uptake of the resulting oligosaccharides provide a strong indication that the microbial population has an active role in the extracellular breakdown of plant cell wall components in the *A. ater* gut.

**Table 4:** Comparison of the glycoside hydrolase (GH) profiles of human, termite, wallaby, giant panda, snail, and slug gut metagenomes as classified by Cardoso et al. (2012a) and Allgaier et al. (2010), showing GH groups that are involved in the breakdown/modification of plant cell wall polysaccharides.

**Pfam group**	**Predominant activity**	**Human**	**Termite**	**Wallaby**	**Panda**	**Snail**	**Slug**
GH5	Cellulases	7	125	27	1	36	15
GH6	Endoglucanases	0	0	0	0	4	0
GH7	Endoglucanases	0	0	0	0	0	0
GH9	Endoglucanases	0	43	5	0	15	11
GH44	Endoglucanases	0	0	0	0	0	0
GH45	Endoglucanases	0	6	0	0	0	0
GH48	Cellobiohydrolases	0	0	0	0	2	0
Total		7	174	32	1	57	26
**ENDOHEMICELLULASES**							
GH8	Endoxylanases	2	21	2	1	46	11
GH10	Endo-1,4-β-xylanase	2	102	19	1	25	16
GH11	Xylanase	0	19	0	0	1	0
GH12	Endoglucanase & xyloglucanase	0	0	0	0	0	12
GH26	β-mannanase & xylanase	1	20	8	0	11	0
GH28	Galacturonases	3	15	10	0	69	6
GH53	Endo-1,4-β-galactanase	11	20	11	4	9	276
Total		19	197	50	6	161	321
**XYLOGLUCANASES**							
GH16	Xyloglucanases	1	6	6	6	12	117
GH17	1,3-β-glucosidases	0	0	0	0	2	60
GH81	1,3-β-glucanases	0	0	0	0	1	0
Total		1	6	6	6	15	177
**DEBRANCHING ENZYMES**							
GH51	α-L-arabinofuranosidases	15	13	19	2	22	3
GH62	α-L-arabinofuranosidases	0	0	0	0	2	0
GH67	α-glucuronidase	1	6	1	2	5	1
GH78	α-L-rhmnosidase	13	7	46	1	73	8
Total		29	26	66	5	102	12
**OLIGOSACCHARIDE DEGRADING ENZYMES**							
GH1	Mainly β-glucosidases	54	27	94	41	294	118
GH2	Mainly β-galactosidases	29	32	39	4	66	60
GH3	Mainly β-glucosidases	55	109	101	11	219	86
GH29	α-L-fucosidases	7	12	5	0	70	11
GH35	β-galactosidase	4	7	8	1	32	14
GH38	α-mannosidase	6	18	3	8	18	39
GH39	β-xylosidase	2	13	3	8	6	279
GH42	β-galactosidases	15	33	17	7	54	6
GH43	Arabinases & xylosidases	34	63	72	13	185	28
GH52	β-xylosidase	0	3	0	0	0	0
Total		206	317	342	93	944	641

Several predicted genes from this metagenome were successfully amplified from whole genome amplified gut metagenomic DNA, confirmed by Sanger sequencing. This validates the assembly and the predictions made thereof, showing that it is very likely that the predicted sequences do exist in nature. We then successfully expressed a full length predicted β-glucosidase gene and observed the enzymatic function using growth plate assays. To our knowledge we are the first to succeed in amplifying novel, functioning genes from a whole genome amplified metagenomic sample. The use of whole genome amplified samples enables studies of a far greater number of predicted genes by sidestepping the problem of small sample size often seen with environmental samples, which limits the scope for genes of interest to be studied using expensive gene synthesis methods.

The use of metagenomics in the study of environmental DNA offers a new means to advance our knowledge of microbial communities. Here we use metagenomics to gain an insight into both the phylogeny and the functional capability of the gut microbiome of the common black slug. This work demonstrates that the microbial community is dominated by a relatively low number of genera with the *Enterobacter* genus being observed in especially high numbers. This study also implicates the slug gut microbiome in the degradation of lignocellulose. Here we identified a large repertoire of genes that offer potential for lignocellulose not only to be degraded but also for the resulting sugars to be taken up by members of the microbiome itself. We have also validated our predictions through amplification of selected glycoside hydrolase genes along with observing predicted functional activity in of an amplified β-glucosidase gene. Our work therefore begins to shed light on how the black slug can process the large quantities of plant biomass it consumes and provides a further example of a herbivore gut microbiome which is well equipped to breakdown plant matter. In addition, by identifying plant pathogen species harbored in the gut we raise questions as to the potential role of the slug in the transmission and wintering of pathogen species. This knowledge is of considerable potential relevance following the 2014 European Union wide ban on the use of some traditional molluscicide pellets in agriculture.

## Data availability

Sequence data from this project has been uploaded to EBI under project number PRJEB21599 (http://www.ebi.ac.uk/ena/data/view/PRJEB21599).

## Ethics statement

As this study uses only invertebrates (*A. ater*), the UK and EU ethics directives for animal testing do not apply. With EU DIRECTIVE 2010/63/EU ON PROTECTION OF ANIMALS USED FOR SCIENTIFIC PURPOSES applying to only “live non-human vertebrate animals” and “live cephalopods.”

## Author contributions

Conceptualization of the project: NF and RJ; Sample collection: RJ; DNA extraction and Molecular Biology: RJ and EO; Bioinformatics analyses: RJ and LP; Data interpretation: RJ and LP; Manuscript writing, RJ and NF with contributions from LP, EO.

### Conflict of interest statement

The authors declare that the research was conducted in the absence of any commercial or financial relationships that could be construed as a potential conflict of interest.

## References

[B1] Agular R. Wink M. (2005). How do slugs cope with toxic alkaloids? Chemoecology 15, 167–177. 10.1007/s00049-005-0309-5

[B2] Allgaier M. Reddy A. Park J. Ivanova N. D'Haeseleer P. Lowry S.. (2010). Targeted discovery of glycoside hydrolases from a switchgrass-adapted compost community. PLoS ONE 5:e8812. 10.1371/journal.pone.0008812 20098679 PMC2809096

[B3] Andrews S. (2010). FastQC A Quality Control tool for High Throughput Sequence Data. Available online at: http://www.bioinformatics.babraham.ac.uk/projects/fastqc/

[B4] Asnicar F. Weingart G. Tickle T. L. Huttenhower C. Segata N. (2015). Compact graphical representation of phylogenetic data and metadata with GraPhlAn. Peer J. 3:e1029. 10.7717/peerj.1029 26157614 PMC4476132

[B5] Aylward F. O. Burnum K. E. Scott J. J. Suen G. Tringe S. G. Adams S. M.. (2012). Metagenomic and metaproteomic insights into bacterial communities in leaf-cutter ant fungus gardens. ISME J. 6, 1688–1701. 10.1038/ismej.2012.10 22378535 PMC3498920

[B6] Bertino-Grimaldi D. Medeiros M. N. Vieira R. P. Cardoso A. M. Turque A. S. Silveira C. B.. (2013). Bacterial community composition shifts in the gut of *Periplaneta americana* fed on different lignocellulosic materials. Springerplus 2:609. 10.1186/2193-1801-2-609 24324923 PMC3855920

[B7] Brune A. (2014). Symbiotic digestion of lignocellulose in termite guts. Nat. Rev. Microbiol. 12, 168–180. 10.1038/nrmicro3182 24487819

[B8] Cardoso A. M. Cavalcante J. J. Cantao M. E. Thompson C. E. Flatschart R. B. Glogauer A.. (2012a). Metagenomic analysis of the microbiota from the crop of an invasive snail reveals a rich reservoir of novel genes. PLoS ONE 7:e48505. 10.1371/journal.pone.0048505 23133637 PMC3486852

[B9] Cardoso A. M. Cavalcante J. J. V. Vieira R. P. Lima J. L. Grieco M. A. B. Clementino M. M.. (2012b). Gut bacterial communities in the giant land snail *Achatina fulica* and their modification by sugarcane-based diet. PLoS ONE 7:e33440. 10.1371/journal.pone.0033440 22438932 PMC3305317

[B10] Dillon R. J. Dillon V. M. (2004). The gut bacteria of insects: nonpathogenic interactions. Annu. Rev. Entomol. 49, 71–92. 10.1146/annurev.ento.49.061802.123416 14651457

[B11] Evans W. A. L. Jones E. G. (1962). A note on the proteinase activity in the alimentary tract of the slug *Arion ater* L. Comp. Biochem. Physiol. 5, 223–225. 10.1016/0010-406X(62)90108-1

[B12] Gismervik K. Bruheim T. Rorvik L. M. Haukeland S. Skaar I. (2014). Invasive slug populations (*Arion vulgaris*) as potential vectors for *Clostridium botulinum*. Acta Vet. Scand. 56:65. 10.1186/s13028-014-0065-z 25277214 PMC4189676

[B13] Hansen A. K. Moran N. A. (2014). The impact of microbial symbionts on host plant utilization by herbivorous insects. Mol. Ecol. 23, 1473–1496. 10.1111/mec.12421 23952067

[B14] Hess M. Sczyrba A. Egan R. Kim T. W. Chokhawala H. Schroth G.. (2011). Metagenomic discovery of biomass-degrading genes and genomes from cow rumen. Science 331, 463–467. 10.1126/science.1200387 21273488

[B15] Huson D. H. Mitra S. Ruscheweyh H. J. Weber N. Schuster S. C. (2011). Integrative analysis of environmental sequences using MEGAN4. Genome Res. 21, 1552–1560. 10.1101/gr.120618.111 21690186 PMC3166839

[B16] Ireland M. P. (1979). Distribution of essential and toxic metals in the Terrestrial gastropod arion-ater. Environ. Pollut. 20, 271–278. 10.1016/0013-9327(79)90150-2

[B17] James R. Nguyen T. Arthur W. Levine K. Williams D. C. (1997). Hydrolase (beta-glucanase, alpha-glucanase, and protease) activity in *Ariolimax columbianus* (banana slug) and *Arion ater* (garden slug). Comp. Biochem. Physiol. B Biochem. Mol. Biol. 118, 275–283. 10.1016/S0305-0491(97)00058-8

[B18] Joynson R. Swamy A. Bou P. A. Chapuis A. Ferry N. (2014). Characterization of cellulolytic activity in the gut of the terrestrial land slug *Arion ater*: biochemical identification of targets for intensive study. Comp. Biochem. Physiol. B Biochem. Mol. Biol. 177–178, 29–35. 10.1016/j.cbpb.2014.08.003 25150536

[B19] Kay P. Grayson R. (2013). Using water industry data to assess the metaldehyde pollution problem. Water Environ. J. 28, 410–417. 10.1111/wej.12056

[B20] Kozlowski J. (2007). The distribution, biology, population dynamics and harmfulness of *Arion lusitanicus* Mabille, 1868 (Gastropoda: Pulmonata: Arionidae) in Poland. J. Plant Protect. Res. 47, 219–230.

[B21] Langmead B. Salzberg S. L. (2012). Fast gapped-read alignment with Bowtie 2. Nat. Methods 9, 357–359. 10.1038/nmeth.1923 22388286 PMC3322381

[B22] Levasseur A. Drula E. Lombard V. Coutinho P. M. Henrissat B. (2013). Expansion of the enzymatic repertoire of the CAZy database to integrate auxiliary redox enzymes. Biotechnol. Biofuels 6:41. 10.1186/1754-6834-6-41 23514094 PMC3620520

[B23] Li H. Handsaker B. Wysoker A. Fennell T. Ruan J. Homer N.. (2009). The sequence alignment/map format and SAMtools. Bioinformatics 25, 2078–2079. 10.1093/bioinformatics/btp352 19505943 PMC2723002

[B24] Lombard V. Ramulu H. G. Drula E. Coutinho P. M. Henrissat B. (2014). The carbohydrate-active enzymes database (CAZy) in 2013. Nucleic Acids Res. 42, D490–D495. 10.1093/nar/gkt1178 24270786 PMC3965031

[B25] Mansfield J. Genin S. Magori S. Citovsky V. Sriariyanum M. Ronald P.. (2012). Top 10 plant pathogenic bacteria in molecular plant pathology. Mol. Plant Pathol. 13, 614–629. 10.1111/j.1364-3703.2012.00804.x 22672649 PMC6638704

[B26] Meyer F. Paarmann D. D'Souza M. Olson R. Glass E. M. Kubal M.. (2008). The metagenomics RAST server - a public resource for the automatic phylogenetic and functional analysis of metagenomes. BMC Bioinformatics 9:386. 10.1186/1471-2105-9-386 18803844 PMC2563014

[B27] Milne I. Stephen G. Bayer M. Cock P. J. A. Pritchard L. Cardle L.. (2013). Using Tablet for visual exploration of second-generation sequencing data. Brief. Bioinformatics 14, 193–202. 10.1093/bib/bbs012 22445902

[B28] Nadarasah G. Stavrinides J. (2011). Insects as alternative hosts for phytopathogenic bacteria. FEMS Microbiol. Rev. 35, 555–575. 10.1111/j.1574-6976.2011.00264.x 21251027

[B29] Namiki T. Hachiya T. Tanaka H. Sakakibara Y. (2012). MetaVelvet: an extension of Velvet assembler to *de novo* metagenome assembly from short sequence reads. Nucleic Acids Res. 40:e155. 10.1093/nar/gks678 22821567 PMC3488206

[B30] Park B. H. Karpinets T. V. Syed M. H. Leuze M. R. Uberbacher E. C. (2010). CAZymes Analysis Toolkit (CAT): web service for searching and analyzing carbohydrate-active enzymes in a newly sequenced organism using CAZy database. Glycobiology 20, 1574–1584. 10.1093/glycob/cwq106 20696711

[B31] Parks D. H. Tyson G. W. Hugenholtz P. Beiko R. G. (2014). STAMP: statistical analysis of taxonomic and functional profiles. Bioinformatics 30, 3123–3124. 10.1093/bioinformatics/btu494 25061070 PMC4609014

[B32] Pope P. B. Mackenzie A. K. Gregor I. Smith W. Sundset M. A. McHardy A. C.. (2012). Metagenomics of the Svalbard reindeer rumen microbiome reveals abundance of polysaccharide utilization loci. PLoS ONE 7:e38571. 10.1371/journal.pone.0038571 22701672 PMC3368933

[B33] Qin J. J. Li R. Q. Raes J. Arumugam M. Burgdorf K. S. Manichanh C.. (2010). A human gut microbial gene catalogue established by metagenomic sequencing. Nature 464, 59–65. 10.1038/nature08821 20203603 PMC3779803

[B34] Russell J. A. Moreau C. S. Goldman-Huertas B. Fujiwara M. Lohman D. J. Pierce N. E. (2009). Bacterial gut symbionts are tightly linked with the evolution of herbivory in ants. Proc. Natl. Acad. Sci. U.S.A. 106, 21236–21241. 10.1073/pnas.0907926106 19948964 PMC2785723

[B35] Sanderson K. (2011). Lignocellulose: a chewy problem. Nature 474, S12–S14. 10.1038/474S012a 21697834

[B36] Segata N. Waldron L. Ballarini A. Narasimhan V. Jousson O. Huttenhower C. (2012). Metagenomic microbial community profiling using unique clade-specific marker genes. Nat. Methods 9, 811–814. 10.1038/nmeth.2066 22688413 PMC3443552

[B37] Seric Jelaska L. Jurasovic J. Brown D. S. Vaughan I. P. Symondson W. O. (2014). Molecular field analysis of trophic relationships in soil-dwelling invertebrates to identify mercury, lead and cadmium transmission through forest ecosystems. Mol. Ecol. 23, 3755–3766. 10.1111/mec.12566 24138157

[B38] Slotsbo S. Hansen L. M. Holmstrup M. (2011). Low temperature survival in different life stages of the Iberian slug, *Arion lusitanicus*. Cryobiology 62, 68–73. 10.1016/j.cryobiol.2010.12.005 21168402

[B39] Suen G. Scott J. J. Aylward F. O. Adams S. M. Tringe S. G. Pinto-Tomás A. A.. (2010). An insect herbivore microbiome with high plant biomass-degrading capacity. PLoS Genet. 6:e1001129. 10.1371/journal.pgen.1001129 20885794 PMC2944797

[B40] Toth I. K. van der Wolf J. M. Saddler G. Lojkowska E. Hélias V. Pirhonen M. (2011). Dickeya species: an emerging problem for potato production in Europe. Plant Pathol. 60, 385–399. 10.1111/j.1365-3059.2011.02427.x

[B41] Zerbino D. R. Birney E. (2008). Velvet: algorithms for *de novo* short read assembly using de Bruijn graphs. Genome Res. 18, 821–829. 10.1101/gr.074492.107 18349386 PMC2336801

[B42] Zhu W. Lomsadze A. Borodovsky M. (2010). Ab initio gene identification in metagenomic sequences. Nucleic Acids Res. 38:e132. 10.1093/nar/gkq275 20403810 PMC2896542

