# Supplementary material for: Metagenomic Analysis of the Gut Microbiome of the Common Black Slug Arion ater in Search of Novel Lignocellulose Degrading Enzymes
Source: Front Microbiol. 2017 Nov 8;8:2181. doi: 10.3389/fmicb.2017.02181 (PMC5682323; doi:10.3389/fmicb.2017.02181)
Supplement: Supplementary file 1 [file DataSheet1.ZIP › supplementary/Supplementary_dataset1.docx]

| **Classification** | **MetaPhlAn percentage abundance** | **MG-RAST percentage abundance** |
| --- | --- | --- |
| **k__Bacteria** | 99.9% | 99.40% |
| **Viruses** | n/a | 0.30% |
| **k__Eukaryota** | n/a | 0.20% |
| **k__Archaea** | 0.01% | 0.10% |
|  |  |  |
| **p__Proteobacteria** | 88.15% | 94.40% |
| **c__Gammaproteobacteria** | 82.16% | 91.27% |
| **o__Enterobacteriales** | 64.56% | 85.30% |
| **f__Enterobacteriaceae** | 64.56% | 85.19% |
| **g__Enterobacter** | 26.86% | 20.20% |
| **g__Citrobacter** | 19.86% | 15.10% |
| **g__Escherichia** | 3.91% | 10.50% |
| **o__Pseudomonadales** | 14.25% | 4.55% |
| **f__Pseudomonadaceae** | 10.56% | 3.40% |
| **g__Pseudomonas** | 10.54% | 3.30% |
| **f__Moraxellaceae** | 3.69% | 1.12% |
| **g__Acinetobacter** | 3.68% | 1.05% |
| **p__Bacteroidetes** | 10.53% | 2.96% |
| **c__Sphingobacteria** | 8.57% | 1.31% |
| **o__Sphingobacteriales** | 8.57% | 1.31% |
| **f__Sphingobacteriaceae** | 8.56% | 1.31% |
| **g__Sphingobacteriaceae_unclassified** | 8.1% | 8.10% |
| **p__Firmicutes** | 0.59% | 1.16% |
| **p__Actinobacteria** | 0.28 | 0.36% |

**Supplementary Dataset 1:** Comparison of phylogenetic analyses using MetaPhlAn (clade specific marker method) and using MG-RAST (best hit based method).
