# Supplementary material for: Metagenomic Analysis of the Gut Microbiome of the Common Black Slug Arion ater in Search of Novel Lignocellulose Degrading Enzymes
Source: Front Microbiol. 2017 Nov 8;8:2181. doi: 10.3389/fmicb.2017.02181 (PMC5682323; doi:10.3389/fmicb.2017.02181)
Supplement: Supplementary file 1 [file DataSheet1.ZIP › supplementary/Supplementary_dataset3.docx]

| **Gene ID number/Primer name** | **Primer sequence** | **Annealing temperature** | **Putative function** | **Predicted size** |
| --- | --- | --- | --- | --- |
| Gene_ID_8282F | ATG TCA CTT ATT CAG AAC CCT G | 53°C | Xylanase | 1680bp |
| Gene_ID_8282R | ATC AAA ACG TGA TTC GCT CGC |  |  |  |
| Gene_ID_71437F | ATG ATG CGT CCA GCC GGT T | 53°C | Cellulase | 1197bp |
| Gene_ID_71437R | TAG CGT GTG ACG GCG CAT |  |  |  |
| Gene_ID_3165F | AGT AAA GAA GCG ATT AAA CGC G | 53°C | FAD-Oxidase (lignin) | 1557bp |
| Gene_ID_3165R | TTG ACC TGC CTG ATG CGA A |  |  |  |
| Gene_ID_77908F | CTC TGG ACG GGC ATG ATG | 57°C | Cellulase | 363bp |
| Gene_ID_77908R | TGA GAA CTT GCG CAT TCC TG |  |  |  |
| Gene_ID_9459F | ATG AGA TAC CGT TTT CCT GAA | 53°C | β-Glucosidase | 1383bp |
| Gene_ID_9459R | ATC GAA TCC ATT ATT GGC GG |  |  |  |
| Gene_ID_13418F | ATG TCC TTG CGT GCT TTA GTC | 57°C | Cellulase | 993bp |
| Gene_ID_13418R | TCA GAC ACC GGT AGC TGC |  |  |  |

**Supplementary Dataset 3:** Primer sequences, predicted function and predicted size of 6 CAZyme gene sequences selected for amplification for metagenome validation
