# Supplementary material for: Metagenomic Analysis of the Gut Microbiome of the Common Black Slug Arion ater in Search of Novel Lignocellulose Degrading Enzymes
Source: Front Microbiol. 2017 Nov 8;8:2181. doi: 10.3389/fmicb.2017.02181 (PMC5682323; doi:10.3389/fmicb.2017.02181)
Supplement: Supplementary file 1 [file DataSheet1.ZIP › supplementary/Supplementary_dataset4.docx]

**Supplementary Dataset 4:** Agarose gel images showing successful amplification of targeted, metagenome predicted, CAZYme genes (yellow markers indicate bands that corresponded to predicted gene sizes and were extracted and sequenced). Supplementary 4D shows successful amplification of the full version of partial gene 77908, after a forward primer was designed based on the top hit of the BLAST output of partial gene 77908 demonstrating the ability to even utilize partial gene sequences for further study through amplification from WGA metagenomic DNA.
