# Supplementary material for: Metagenomic Analysis of the Gut Microbiome of the Common Black Slug Arion ater in Search of Novel Lignocellulose Degrading Enzymes
Source: Front Microbiol. 2017 Nov 8;8:2181. doi: 10.3389/fmicb.2017.02181 (PMC5682323; doi:10.3389/fmicb.2017.02181)
Supplement: Supplementary file 1 [file DataSheet1.ZIP › supplementary/Supplementary_dataset5.docx]

**Supplementary Dataset 5:** Nucleotide sequences for genes amplified as part of metagenome validation steps

**>gene_id_9459_nucleotide**

ATGAGATACCGTTTTCCTGAAAACTTCTGGTGGGGCAGTGCCTGCTCAGCGCTGCAAACCGAAGGGGATAGCCTGAACGGCGGTAAAAGCCAGACCACATGGGACGTGTGGTTCGACCGTCAGCCAGGTCGTTTCCATCAGGGGGTTGGCCCGGCAGACACCTCCGGTTTTTATCAACACTGGAAACAGGACATCGCACTGCTGAAACAGCTACAGCACAACAGCTTTCGTACATCCTTGAGCTGGTCGCGTCTGATCCCGGACGGCACCGGCGAGGTCAATCCCGAAGCGGTCGATTTCTATAACAACGTCATCGACGAGCTGCTGGCGCAGGGTATCACGCCCTTTATCACCCTGTTTCACTTCGATATGCCAATGGTCATGCAGGAGAAAGGCGGCTGGGAAAATCGTGAAGTTGTGGAAGCCTTTGGCCGCTACGCCCAGACGTGTTTTACCTTGTTCGGCAATCGGGTGAAGCACTGGTTCACCTTCAACGAACCGATTGTCCCGGTAGAAGGCGGCTATCTGTATGACTTCCACTACCCGAACGTGGTGGATTTCAAACGTGCGGCAACCGTGGCTTACCACACCGTACTGGCGCATTCGACGGCGGTCCGTGCGTATCGCGCTGGCAACTATGACGGTGAAATCGGCGTGGTGCTGAATTTAACGCCGTCTTATCCGCGCTCGCAAAATCCGGCGGATGTGAAAGCCGCGCACCATGCGGATCTGCTGTTTAACCGCAGCTTCCTCGATCCGGTATTAAAAGGCGAATATCCGGCGGACCTGGTGGAACTGTTGAAACAGTACGATCAACTACCGGCCTGCCAGCCCGGCGACAGCCAGCTCATCGCCGAGGGTAAAATCGACCTGTTGGGCATTAACTACTACCAACCACGCCGGGTGAAATGCCGCGATAGCGCCGTCAATCCTGACGCGCCGTTTATGCCAGAGTCGCTGTTTGATTATTACGAAATGCCGGGACGTAAAATGAATCCGTACCGTGGCTGGGAAATCTATGAGCCGGGCATTTACGATATTATTACGAATCTGCGTGATAATTATGGCAACCCGCGCTGTTTTATTTCTGAAAACGGCATGGGTGTAGAAAACGAGCAGCGTTTTATTCAGGACGATCAGATTAACGACAATTACCGTATTGAGTTTGTCTCGGAACATCTTAAATGGCTGCATAAAGGCATTAACGAGGGTTGTCATTGCCTTGGTTACCATATGTGGACCTTTATTGATAATTGGTCATGGCTTAACGGATATAAAAACCGCTACGGTTTTGTCCAGTTAGATTTAGCCACGCAAAAACGTACAGTGAAAAAGAGCGGCGAGTGGTTTGCGAAAACTGCCGCCAATAATGGATTCGATTAA

**>gene_id_13418_nucleotide**

ATGTCCTTGCGTGCTTTAGTCGCAGTCATGGTTACGACGACGATGATGGTGCTTCCCCGCGCGTGGGCAGATACCGCCTGGGAAAGCTATAAATCCCGTTTTATGATGGCCGATGGGCGAATCGTTGATACCGGCAACGGCAGCGTTTCCCATACCGAAGGGCAGGGGTTCGCCATGCTGCTGGCGGTCGCCAAAGACGATCGCCCTGCCTTTGATAAGCTGTGGCAGTGGACGGATAAAACGCTGCGTAATAAAGACAACGGGCTATTTTACTGGCGCTACAACCCCGTCGCGCCGGACCCTATTGCGGATAAAAACGATGCGACCGACGGCGATTCCCTGATCGCGTGGGCGCTGCTACGCGCCCAACAGCAGTGGGGTGATAAATCCTACGGCATCGCTTCAGATGCCATTACCGCGTCGCTGCTGAAGTCTACGGTCATCACCTTTGCCGGTCATCAGGTGATGCTGCCCGGTGCGAAGGGCTTCAACCGCAATGATCATGTCAACCTCAATCCCTCCTACTTTATTTTCCCCGCATGGCAGGCCTTTGCCGCGCGTACGCACCTGACGGCGTGGCGTAAGCTGCAGAGCGACGGGAAGGCGCTGCTGGGTAAAATGGCGTGGGGTAAGGCGCAGCTGCCCAGCGATTGGGTGGCGCTGAGAGCGGACGGCAGAATGGAGCCGGCAAAGGAGTGGCCGCCCCGGATGAGCTACGACGCGATTCGCATCCCGCTTTATCTCTCCTGGGCCGATCCGCAAAGCGCCCTGCTGACGCCGTGGAAAAGCTGGTTTCAGAGCTATCCGCGGCTGCAGACTCCGGCGTGGGTCAACGTCAATACCAACGACGTGGCCCCGTGGTTTATGACCGGCGGCCTGCTGGCCGTTCGCGACCTGACCACCGGAGAAGCACAGGACGATCCGCAGCTTAGCGCGCAGGATGACTATTACTCTGCCAGCCTGAAGATGCTGGTCTGGCTGGCGAAGAACGACCGCCGCCAGGCAGCTACCGGTGTCTGA

**>gene_id_77908_nucleotide**

CTCTGGACGGGCATGATGCACGACCGCGATCCGCAAAAAGCCCGACTGCTGGCACGTTTTAAACCGATGGCGACGCTCACAACAAAAAATGGCGTCCCGCCGGAGAAGGTCGATGTCACAAGCGGTAAACCCACGGGCGATGGCCCGGTAGGCTTTTCCGCCTCGCTGCTGCCGTTTTTACAGGACCGCGATGCACAAGCGGTGCAACGCCAGCGCGTCGCCGACCATTTCCCCGGCAATGATGCCTATTACAGCTACGTGCTGACCCTGTTCGGACAAGGATGGGATCAGCACCGTTTTCGCTTTACCGCAAAGGGTGAATTACACCCTGACTGGGGCCAGGAATGCGCAAGTTCTCATTAA

**>gene_id_71437_nucleotide**

ATGATGCGTCCAGCCGGTTGGGTTGCGCTGGGCATCGCGCTCTTCGCCGGCGCAGCCCAGGCGCAGACGTGCGACGCGCAGTGGCCACTGTGGCAGAACTACGCGAAGCGGTTTGTGCAGGATGACGGGCGGGTGCTGAATTCGTCCCTGAACCCCAGCGAAAGCAATTCTGAAGGACAGTCCTACGCGATGTTTTTTGCGCTGGTGGGCAACGACCGCGCGCGGTTTGACAAGCTCTGGACCTGGACCAAGGCCAATATGGCCGGCAACGACATCAGCCGCACCCTGCCGGGTTGGTTGTGGGGCAAGACCCAGAGCGGCGAATGGGGCCTGATCGACGCCAATTCCGCCAGCGATGCTGACCTGTGGGTCGCCTACGCGCTGCTGGAAGCGGCACGTGTATGGAATGTGCCGCAGTACCGCGCCGATGCGCAGTTGGTGTTGGCCAATGTCGAAAAGACCTTGATCGTACGCGTGCCGGGCCTGGGCAAGATGCTGTTGCCGGGGCCGGTGGGTTACAGCTACCCAGACGGTTTGTGGCGTTTCAACCCCAGCTACCAAGTGCTGGCGCAACTGCGACGCTTCCACAAAGAACGCCCCAATGGCGGCTGGAATGAGGTGGCTGAGAGCAACGCCAAAATGCTCGCCGACCCCAAGAGCAACCCCCATGGCATCGCCGCCAACTGGGTGGGTTACCGCGCCACGGGTGCAAACACCGGGGTGTTTGTGGTCGATCCGTATTCCGATGACCTGGGCAGCTACGACGCCATCCGCACTTACCTGTGGGCTGGAATGACCGCCAAAGGGGACCCGCTGGCGGCGCCGATGCTCAAGGCGTTGGGTGGTTTTTCGCGTGCTACGGCGGCGTCTCCCAATGGCTTGCCACCGGAGAAGATTCACGTGCTCACCGGTGTGGCCGAAAAGAACAACGGCTATTCGCCGCTGGGGTTCTCGGCATCTGCTCTGGTGTTCTTCCAGGCACGCGGCGAAACCGCCCTGGCTCAACTGCAAAAACACAAGCTGGATGATGTGCTGGGCAAGGCTCTGGTCGCATCAGCGGCCGACGGCGATCAGCCGGTGTATTACGACTACATGCTCAGCCTGTTCAGCCAAGGCTTTACCGATCAAAAGTACCGCTTCGAACAGGACGGTACGGTCAAATTATTCTGGGAGGGCGCATGCGCCGTCACACGCTAG

**>gene_id_8282_nucleotide**

ATGTCACTTATTCAGAACCCTGTACTCCCTGGTTTTAACCCAGACCCAAGCATTATCCGTGTAGAAGATACCTACTATATCGCAAACTCAACGTTTGAGTGGTTCCCTGGCGTTCGCTTGCATGAATCAAAAGACCTGCAAAACTGGACTCTTCTGCCCAGTCCTCTGTCCACAACAGCGCTTTTAGATATGAAAGGCAACCCCTCTTCTGGCGGGATTTGGGCTCCGGCGCTCTCATACGCCGATGGCAAATTCTGGTTGGTTTATACAGATGTGAAAATCACAGAAGGCGCTTTTAAGGACATGACAAATTATCTGACCACCGCAACAGATATACGTGGTCCGTGGACCGATCCCATCAAACTAAATGGCGTCGGCTTTGATGCGTCACTGTTCCATGATGAAGACGGGCGTAAATATTTGGTTCAACAAACCTGGGACCATCGCGAATACCATCATCCTTTTGATGGTATCACCTTAACGGAGCTGGATACCCGGACCTTAAAATTAAAACCAGAGACAGCGCGTACGATTTATCGCGGCACTGCCGTTGCACTTGTTGAGGGGCCACATCTCTACAAGCTGAACGGGTACTACTATCTTTTCGCCGCTCAGGGCGGGACTGTATTTACCCACCAGGAGGTGGTTGCGCGATCCAGAACCTTAGAGGCCAACAGCTTTGAAACACAACCGGGAGAGGTATTCTTAACTAACGTCGATACGCCAGACAGCTATATCCAGAAGCAGGGGCATGGGGCGTTGGTGTCCACCCCCAGCGGTGAGTGGTATTATGCCTCGCTCTGCGCACGTCCGTGGAATCGTGCAGGTGAGTCAGCCTACGATCCTCGTGGTTGGTCAACCCTTGGCCGGGAAACGTCTATTCAGAAAGTGTACTGGGATGAAGATGGCTGGCCACGTATTGCGGGAGGCCATGGTGGAAAAACCTTTGTCGAGGGGCCGGCCGATGCCATTTATACCGAAAGCGCAAAAGACCATAGCCAGCACGATGATTTTAAAACGGCGACACTGGATATTAACTGGAATACGCTTCGTGTCCCCTTTACAGAAAAAATGGGTACCACGGGCGATGGAAGGCTGACGTTAACGGGACAGGGTTCTTTAGCGAATACCCATAACCTTTCGCTGATTGCCCGACGCTGGCAAGCCTTTTATTTTGATGCTCAGGTTAAAGTCAAATTTAATCCATTTAATTACCAACAAATGGCCGGGTTGACGAATTATTATAATGACCGCCACTGGAGTTTCGTTTTCATTACCTGGAATGAAATCAACGGCTCTGTCATCGAAATAGGTGAGAATAACCGTGGAAAATATACCTCTTATTTGAAAGATAACGCCATCAAGATCCCAGAGGGCACAGAATTTGTCTGGTTCCGCACGAAAGTTCGGAAGGAAACTTATACGTATGAATACAGCTTCGATAGCATCACTTTCACAGAGATCCCTGTTAAATTAGATGCCGCTATTCTTTCTGATGATTATGTCCTGCAAAGCTACGGCGGCTTCTTTACCGGAGCGTTCGTTGGCCTGGCGGCAGTCGACTACTCTGGTTACGATGCCAGCGCAGAGTTTTATAACTTTGTTTATCAGGAGCTTGGCGATAAGAAAACTGGCGATAATGCCTGGAGCTGGGACGCGAGCGAATCACGTTTTGATTAA

**>gene_id_3165_nucleotide**

ATGATCAGTAAAGAAGCGATTAAACGCGGTTACAACCGTGGAAATTATACGGTAGGTGCACACACGCGCCCGGCATGGGCGACGGATATCAAAAGCAGCGGTGGCGCGAAAGGGATGCAGATTGATCCGGTGGCGATGAGCGAGGCAACGTTTGACCGGCTGCGGGTCATTGCTGATGAGGTGCTGACGTCGGAGTCTGATGTGATTACCTGGACCCGCGACTGGTGGGCCGGATCGATGATTGCGGAAACCCAGGGCGCACCGGCCACGTCAAAAGGGGCGATTGTACGTGTCTCTACGGTAGAACAAATTCAGGATGTGATGCGTCTGGCGAATGCGTTGGCAATCCCGGTCACGGTTTCCGCCGGGCGCAGTAACGTCACTGGCGCCGCATTGCCGTTGCGGGGGGGCATTGTGCTGGATGTTTGCGAACTCAACAAACTTATCGACTTCGATCCACAAAGTCAGATTGTTGACGTTGAAGCCGGCATGTTCGGCGATATTTTTGAACAAACTATCCAAAAAGAGTACGGCATGACGATGGGCCACTGGCCGTCATCGTTTGGTATCAGTACCGTGGGCGGTTGGGTGGCCTGCCGTGGCGCGGGACAGTTGTCGACCCGCTACGGCAAAATTGAAGATATGGTTTTTGGGATGGATGTTGTCCTTGCCGATGGCCGCTTAATCACCGTGGGTGGCTATGCGCGTAGCGCAACGGGGCCTGATCTACAGCAGATGTTTATTGGTTCTGAAGGCACATTGGGGGTGATTGTCCGTGTGCGTCTGAAACTGCATCGTTTACCTGACTATGGGCGTGCCATCGCCTGGGGTTTTTCCAGCTTCGCCAGTGGACTGGAGGCATGCCGTGAAATTTTACAGCATGGCGCGAACCCCGCAGCGTTGCGCCTGTACGATAACCTGGAAAGCGGCGTGCAGTTTGGTTTACCAGATACCAATGTCCTGTTGATTGCGGATGAGGGGGAGCCAGAGATTGTCGATGCGGTGCTGGCTATCAGCGAACGTGTGTGCCAACGCAGCGGACAACAACTCGACGGCGAGGCTATTTTTGAACGCTGGCTGGACACCCGATATCTGACCGGAAAAAGTGCTGAAGGCTTTAAAAAGAGCCCGGGTCTGGTGGCGGATACGCTGGAAATGGTGGGACGTTGGCGCGATCTTAGCGACGTGTATGATGATGTGGTGGCTGCGATTAATGCCGTACCCGGCACGCTGGCCGGTTCGGCGCATCAGTCTCACGCCTACATCGACGGCGCATGCCTCTATTTCTCATTGCGCGGTGATGTGGCGATTGAGCAACGTGCGGCCTGGTATCGTGCGGCATGGGATGCAGCGAATGCGGTGCTAATTAAGCACAATACCTCCCTTAGCCACCATCATGGTGTTGGTCTCCTGCGTTCGCCCTATATGCAGGCTTCTTTAGGCGAATCGCTTTCCGTTCTGGCGGATATCAAATATGCGCTGGATCCGAAAAACATTCTTAACCCAGGCAAGCTGGGGTTAAGCGTCGATCTTCCTTCGCATCAGGCAGGTCAATAA

**>gene_id_77908full_nucleotide**

ATGGTCGCGCTGGTTCTGGCGGCAGCGAATGCGCGTGCGGCCTGTAGCTGGCCCGCGTGGGAGCAGTTTAAACAGGACTACATCAGCGATGGCGGGCGCGTGATTGATCCCAGTGACGCGCGGAAAATCAGCACTTCGGAAGGGCAAAGCTATGCGCTGTTCTTTGCCCTGGCCGCCAACGATCGCAAAGCGTTCGATTTACTGCTGACCTGGACGAGCGACAATCTCGCCCAGGGCTCCCTGAGTCAGCATCTGCCTGCCTGGTTGTGGGGGAAAAAGGATGCGGATACCTGGGCGGTGATCGACAAAAACTCTGCGTCTGATGCGGATATCTGGATTGCCTGGTCGTTGCTGGAAGCGGGGCGTTTGTGGAAAGCGCCGCAATACACCGCCACCGGCAAAGCACTGCTAAAACGCATCGCCAGCGAAGAAGTGATCAAAGTGCCGGGTTTAGGGCTGATGCTCCTGCCCGGCAACGTCGGTTTTACCGAGGAGAAAGCCTGGCGCTTTAACCCCAGCTATCTCCCGCCGCAGCTGGCGAACTATTTCACCCGCTTTGGCGCGCCGTGGACCACGCTTCGCGAGACGAATCTGCGTTTACTGCTGGAAACCGCGCCAAAAGGATTTGCGCCCAACTGGGTGCAGTATCAGCAAAAAAAAGGCTGGCAATTGCAGCCAGAAAAAACCTTTATCGGCAGTTACGACGCGATTCGCGTGTATCTCTGGACGGGCATGATGCACGACCGCGATCCGCAAAAAGCCCGACTGCTGGCACGTTTTAAACCGATGGCGACGCTCACAACAAAAAATGGCGTCCCGCCGGAGAAGGTCGATGTCGCAAGCGGTAAACCCACAGGCGATGGCCCGGTCGGTTTCTCCGCCTCGCTGCTGCCTTTTTTACAGGACCGTGATGCACAAGCGGTGCAACGCCAGCGCGTCGCCGACCATTTTCCCGGCAATGACGCCTATTACAGCTACGTGCTGACCCTGTTCGGACAAGGATGGGATCAGCATCGTTTTCGCTTCACCGCAAAGGGTGAATTACACCCTGACTGGGGCCAGGAATGCGCAAGTTCTCATTAA
